# Supplementary material for: Causal Relationship between Aging and Anorexia Nervosa: A White-Matter-Microstructure-Mediated Mendelian Randomization Analysis
Source: Biomedicines. 2024 Aug 16;12(8):1874. doi: 10.3390/biomedicines12081874 (PMC11351342; doi:10.3390/biomedicines12081874)
Supplement: Supplementary file 1 [file biomedicines-12-01874-s001.zip › Supplementary Figure/Figure S4/Singe SNP plot of MR analysis for TL to ubm-b-1585.pdf]

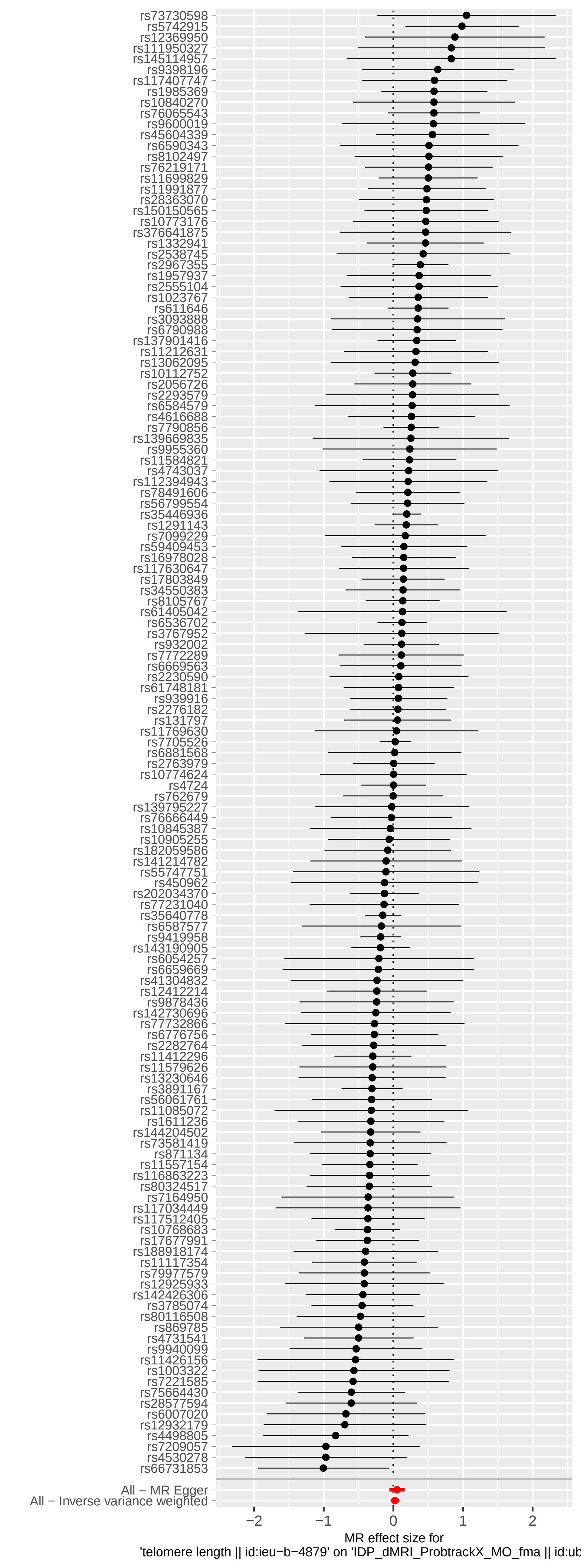
rs73730598
rs5742915
rs12369950
rs111950327
rs145114957
rs9398196
rs117407747
rs1985369
rs10840270
rs76065543
rs9600019
rs45604339
rs6590343
rs8102497
rs76219171
rs11699829
rs11991877
rs28363070
rs150150565
rs10773176
rs376641875
rs1332941
rs2538745
rs2967355
rs1957937
rs2555104
rs1023767
rs611646
rs3093888
rs6790988
rs137901416
rs11212631
rs13062095
rs10112752
rs2056726
rs2293579
rs6584579
rs4616688
rs7790856
rs139669835
rs9955360
rs11584821
rs4743037
rs112394943
rs78491606
rs56799554
rs35446936
rs1291143
rs7099229
rs59409453
rs16978028
rs117630647
rs17803849
rs34550383
rs8105767
rs61405042
rs6536702
rs3767952
rs932002
rs7772289
rs6669563
rs2230590
rs61748181
rs939916
rs2276182
rs131797
rs11769630
rs7705526
rs6881568
rs2763979
rs10774624
rs4724
rs762679
rs139795227
rs76666449
rs10845387
rs10905255
rs182059586
rs141214782
rs55747751
rs450962
rs202034370
rs77231040
rs35640778
rs6587577
rs9419958
rs143190905
rs6054257
rs6659669
rs41304832
rs12412214
rs9878436
rs142730696
rs77732866
rs6776756
rs2282764
rs11412296
rs11579626
rs13230646
rs3891167
rs56061761
rs11085072
rs1611236
rs144204502
rs73581419
rs871134
rs11557154
rs116863223
rs80324517
rs7164950
rs117034449
rs117512405
rs10768683
rs17677991
rs188918174
rs11117354
rs79977579
rs12925933
rs142426306
rs3785074
rs80116508
rs869785
rs4731541
rs9940099
rs11426156
rs1003322
rs7221585
rs75664430
rs28577594
rs6007020
rs12932179
rs4498805
rs7209057
rs4530278
rs66731853
All − MR Egger
All − Inverse variance weighted
−2
−1
0
1
2
MR effect size for
'telomere length || id:ieu−b−4879' on 'IDP_dMRI_ProbtrackX_MO_fma || id:ub
